# Evaluation of sequencing and PCR-based methods for the quantification of the viral genome formula

**DOI:** 10.1016/j.virusres.2023.199064

**Published:** 2023-02-07

**Authors:** Dieke Boezen, Marcelle L Johnson, Alexey A Grum-Grzhimaylo, René AA van der Vlugt, Mark P Zwart

**Affiliations:** a Netherlands Institute of Ecology (NIOO-KNAW), Droevendaalsesteeg 10, Wageningen 6708PB, The Netherlands; b Laboratory of Virology, Wageningen University, Droevendaalsesteeg 1, Wageningen 6708PB, The Netherlands; c Westerdijk Fungal Biodiversity Institute, Uppsalalaan 8, Utrecht 3584CT, The Netherlands

**Keywords:** Multipartite virus, Genome formula, Cucumber mosaic virus, Quantitative PCR, Digital PCR, Long-read sequencing

## Abstract

Viruses show great diversity in their genome organization. Multipartite viruses package their genome segments into separate particles, most or all of which are required to initiate infection in the host cell. The benefits of such seemingly inefficient genome organization are not well understood. One hypothesised benefit of multipartition is that it allows for flexible changes in gene expression by altering the frequency of each genome segment in different environments, such as encountering different host species. The ratio of the frequency of segments is termed the genome formula (GF). Thus far, formal studies quantifying the GF have been performed for well-characterised virus-host systems in experimental settings using RT-qPCR. However, to understand GF variation in natural populations or novel virus-host systems, a comparison of several methods for GF estimation including high-throughput sequencing (HTS) based methods is needed. Currently, it is unclear how HTS-methods compare a golden standard, such as RT-qPCR. Here we show a comparison of multiple GF quantification methods (RT-qPCR, RT-digital PCR, Illumina RNAseq and Nanopore direct RNA sequencing) using three host plants (*Nicotiana tabacum, Nicotiana benthamiana*, and *Chenopodium quinoa*) infected with cucumber mosaic virus (CMV), a tripartite RNA virus. Our results show that all methods give roughly similar results, though there is a significant method effect on genome formula estimates. While the RT-qPCR and RT-dPCR GF estimates are congruent, the GF estimates from HTS methods deviate from those found with PCR. Our findings emphasize the need to tailor the GF quantification method to the experimental aim, and highlight that it may not be possible to compare HTS and PCR-based methods directly. The difference in results between PCR-based methods and HTS highlights that the choice of quantification technique is not trivial.

## Highlights

• The genome formula of CMV is the same for *N. tabacum, N. benthamiana* and *C. quinoa*.
• The choice of genome formula (GF) detection method affects the GF estimate.
• PCR-based estimates of the GF are not fully congruent with HTS-based GF estimates.

## 1 Introduction

Viral genomes can be organised and packaged in several ways, consisting of three main modes of packaging. Monopartite viruses consist of a single genome segment packaged in a single particle. Segmented viruses have multiple genomic segments which are packaged in a single viral particle. Multipartite viruses have a genome consisting of multiple segments, each packaged independently.

Plants are the most common host of multipartite viruses (Lucía-Sanz et al., 2018), though multipartite viruses have also been detected in insects (Hu et al., 2016; Ladner et al., 2016). In multipartite viruses, the different genome segments accumulate at a ratio which, counterintuitively, is not equal per segment. The frequency of segments after infection converges on an equilibrium, regardless of their frequency at the start of the infection (Wu et al., 2017; Sicard et al., 2013). This equilibrium has been shown to be host dependent (Sicard et al., 2013). The set of relative frequencies of the different virus segments is referred to as the genome formula (GF) or, when at the host-specific equilibrium, setpoint genome formula (SGF) (Sicard et al., 2013).

GF adjustment is one of the hypothesized benefits of the otherwise costly multipartite genome organization (Sicard et al., 2016). The cost of multipartition arises from having independently packaged viral genome segments, which requires a higher multiplicity of infection (MOI) for between-host transmission. Modeling work has shown a relationship between MOI and the GF (Zwart and Elena, 2020). Under highly heterogeneous environments, a multipartite virus can have the advantage of rapidly tuning gene expression through shifts in the GF (Zwart and Elena, 2020; Sicard et al., 2013). Data on variation in GFs across environments is sparse, but is known to be dependent on the host species (Sicard et al., 2013; Wu et al., 2017), host tissue type (Hu et al., 2016; Dall’Ara, 2018), and presence of satellite viruses (Obrępalska-Stęplowska et al., 2015; He et al., 2019; Mansourpour et al., 2022). Although the conceptual framework of the GF as an adaptive strategy arose in the context of multipartite evolution, evidence of unequal accumulation of segments has also been observed in segmented viruses (Moreau et al., 2020; Diefenbacher et al., 2018). Reliable GF estimation is thus important for identifying evolutionary benefits of multipartition.

Much of the work focused on the GF has been done using model virus-host systems under controlled laboratory conditions with qPCR-based estimation of the GF (Wu et al., 2017; Sicard et al., 2013) (Table 1). Though qPCR can reproducibly estimate the GF, there are several disadvantages to this method. Firstly, when estimating the GF with qPCR there is limited resolution with which the GF can be reported (e.g. a GF estimation of 1:3:2 vs 9:32:19) due to variation between technical replicates. When small GF differences are biologically relevant, other methods might be more appropriate. It is not uncommon for published qPCR assays not to be fully validated or reported according to the MIQE guidelines (Bustin et al., 2009). Secondly, the use of PCR-based methods is sequence-specific requiring careful primer design and further testing to ensure segment frequencies can be precisely and accurately determined. Within model virus-host systems, this is not a major limitation as often whole genome sequence data is available. However, for large surveys of ecosystems containing novel variants, primer design and testing is not feasible due to the quantity of targets to assay and the time and investment in RT-qPCR development. Although qPCR is considered the ‘gold-standard’ for DNA/RNA quantification, alternative techniques exist which may improve upon its precision and mitigate bias.

**Table 1:** Genome formula (GF) studies and techniques used for GF quantification.

Virus species	Baltimore classification	Number of genomic segments	Technique used for quantification of GF	Refs.
Faba bean necrotic stunt virus (FBNSV)	ssDNA	8	qPCR	(Sicard et al., 2013)
Faba bean necrotic stunt virus (FBNSV)	ssDNA	8	qPCR, Illumina	(Gallet et al., 2017)
Alfalfa mosaic virus (AMV)	(+)ssRNA	3	qPCR	(Wu et al., 2017)
Bombyx mori bidensovirus (BmBDV)	ssDNA	2	qPCR	(Hu et al., 2016)
Banana bunchy top virus (BBTV)	ssDNA	6	qPCR, Illumina	(Yu et al., 2019)
Rice stripe virus (RSV)	(-)ssRNA	4	qPCR	(Zhao et al., 2019)

Digital PCR (dPCR) allows for absolute quantification of DNA/RNA by dividing a single PCR reaction into thousands of small partitions in which separate reactions take place (Vogelstein and Kinzler, 1999). Due to mixing prior to partitioning, each partition contains PCR reaction mixture and a small amount of the template added. These partitions can be droplets or physical partitions on a microfluidic chip. Using Poisson statistics on the frequency of negative partitions and data on partition volume, a precise estimation can be made of the number of target copies in the sample (Kreutz et al., 2011). Thus, the error margin on a dPCR estimate is narrower than for qPCR estimates, and any PCR inhibitors present in the sample will not have a major effect on quantification (Rački et al., 2014). dPCR has been tested for the detection and quantification of pathogens in human health (Ma et al., 2013), infectious disease (Hayden et al., 2013; Pholwat et al., 2013), environmental microbiology (Tadmor et al., 2011; Morella et al., 2018) and for monitoring in wastewater samples (Stachler et al., 2019). In plant virology it is used for detection of regulated viruses (Gutiérrez-Aguirre et al., 2015) and differentiation between closely related virus genotypes (Mehle et al., 2020). dPCR is a promising method for precise estimation of the GF, such as for the quantification of GF changes following experimental evolution or to determine the frequency of variants over time. As with qPCR-based GF estimation, dPCR requires the design and testing of primers, and is therefore not applicable to quantification of novel viruses.

High-Throughput Sequencing (HTS) methods enable a less biased assessment of all genetic material, including any virus in a sample, and allow for GF estimation based on the number of reads that map to a virus segment. Illumina sequencing is an established method for the detection of (plant) viruses, both in (agro)ecosystems and phytosanitary screening of crops (Hasiów-Jaroszewska et al., 2021; Shates et al., 2018; Fetters et al., 2022; Hammond et al., 2021; Maclot et al., 2020). Illumina sequencing generates short DNA or cDNA reads of 100–300 bp. When mapping these reads to a reference gene or genome, the coverage across this region is indicative of its relative accumulation or ‘expression’. Due to variation in GC-content, coverage is often uneven across the genome when using short-read sequencing platforms (Benjamini and Speed, 2012; Dohm et al., 2008).

Long-read sequencing (LRS) approaches generate long reads (>500 bp). Currently, two platforms are widely used: the Pacific Biosciences (PacBio) HiFi platform and the Oxford Nanopore Technologies (ONT) MinION and GridION platforms. Long-read sequencing often enables the sequencing of a full virus genome segment in a single read (Wongsurawat et al., 2019). Single stranded RNA can be sequenced directly (dRNA sequencing) without the need for an amplification step using Nanopore sequencing, thus avoiding PCR bias. Long-read sequencing is a relatively new technique which has been used for (plant) virus detection (Liefting et al., 2021; Bronzato Badial et al., 2018; Filloux et al., 2018; Fellers et al., 2019; Phannareth et al., 2021). Longer read length results in a more even coverage across the genome compared to short reads. This should allow for a reasonably unbiased estimation of each multipartite virus segment. Nanopore sequencing has been used for the identification of a novel bipartite ssDNA begomovirus (Martins et al., 2021) and CRESS viruses, a group of putative multipartite ssDNA viruses (Ben Chehida et al., 2021), multipartite virus alfalfa mosaic virus (AMV) and a segmented RNA virus tomato spotted wilt virus (TSWV) (Liefting et al., 2021). A drawback of dRNA Nanopore sequencing is that it does not allow for multiplexing different samples in a single run, making the technique more laborious than the Illumina RNA sequencing platform.

In this study, we experimentally evaluate the four above-mentioned methodological approaches for quantifying the GF of cucumber mosaic virus (CMV), a tripartite positive-sense single-stranded RNA virus. We test whether the results are congruent and provide guidelines for when each approach is most suitable. We compare GF measurements from RT-qPCR, RT-dPCR, and HTS-based methods: Illumina sequencing (short-read sequencing) and Nanopore sequencing (long-read sequencing). We show systematic, method-specific biases for GF estimation, and that qPCR and dPCR methods are highly congruent in their GF estimates, as are the HTS-based estimates (Nanopore and Illumina).

## 2 Methods

### 2.1 Infection of plants with CMV

Ten replicate *Chenopodium quinoa, Nicotiana tabacum* and *Nicotiana benthamiana* plants were inoculated with cucumber mosaic virus isolate I17F (Subgroup I) (Jacquemond and Lot, 1981) (CMV I17F, hereafter referred to as “CMV”, obtained from the plant virus collection of Wageningen University and Research) by mechanical inoculation as follows. Frozen *N. tabacum* tissue previously infected with CMV was homogenised in phosphate inoculation buffer (a 49:51 mix of two solutions: 1.362 g KH_2_PO_4_ dissolved in 1 liter deionized water and 1.781 g Na_2_HPO_4_·2H_2_O dissolved in 1 liter deionized water, respectively. 0.01 M, pH 7) (Hull, 2009). Carborundum powder was dusted onto the youngest leaf, and 30μl of the homogenised inoculum was rubbed onto the leaf and rinsed with water shortly after. Similarly, three plants for each species were mock-inoculated using only phosphate inoculation buffer. Plants were kept in the greenhouse (day length of 16 h, temperature 22 °C day/18 °C night, 60% humidity) and were watered 25 ml every two days for the duration of the experiment.

After eight days (*C. quinoa*) or fourteen days (*N. tabacum, N. benthamiana*), leaves were monitored for symptoms and harvested. For *C. quinoa* - a local lesion host - the inoculated leaf with developed lesions was harvested. For *N. tabacum* and *N. benthamiana* the second youngest leaves (upper uninoculated leaves) were harvested. Sampled leaves were stored in sterile homogenisation bags (BioReba) and stored at −80 °C immediately after harvesting.

### 2.2 CMV detection in inoculated plants

Leaves were homogenised in 2 ml extraction buffer A (BioReba) using a handheld homogeniser. 50 μl of the homogenised tissue was diluted in 150 μl extraction buffer A and CMV infection was confirmed by an antibody assay (Agristip, Bioreba). Samples which tested positive in the diagnostic assay were immediately used for RNA extraction.

### 2.3 RNA extractions

For each plant species, four infected samples and one mock inoculated sample were randomly selected out of ten replicates for total RNA extraction and downstream analysis using the different quantification techniques. For *N. tabacum*, RNA was extracted from 100 μl of homogenised leaf tissue using the RNeasy Plant Mini Kit (Qiagen), following the manufacturer's protocol (with on-column DNAse digestion). For *C. quinoa*, RNA was extracted from 200 μl of homogenised leaf tissue using the Direct-zol RNA miniprep kit (Zymo), following the manufacturer's protocol. For *N. benthamiana*, RNA was extracted from 100 μl or 200 μl of homogenised leaf tissue using the Direct-zol RNA miniprep kit (Zymo), following the manufacturer's protocol. For all samples, RNA was eluted in 40 μl RNase free water, and yield and purity were determined using the Nanodrop spectrophotometer and Qubit3 fluorometer (RNA HS Assay kit, Invitrogen). After extraction, multiple aliquots of RNA were stored at −80 °C.

### 2.4 Quantitative PCR

In preparation of qPCR and dPCR, 250 ng of total RNA was converted to cDNA using iScript Reverse Transcription Supermix (BioRad), which uses a combination of oligo(dT) and random hexamers. CMV segments were quantified using a SYBR-based qPCR assay (BioRad) using three mastermixes: one for each of the three RNA segments. cDNA samples were diluted 100-fold and assayed in triplicate for RNA1, RNA2 and RNA3 each. Primer sequences can be found in Table S2. Thermocycling consisted of initial denaturation at 95 °C for 3 min, followed by forty cycles of 95 °C for 10 s and 60 °C for 30 s, and a final step consisting of 95 °C for 10 s and a melt curve ranging from 65 °C to 95 °C in 0.5 °C increments. The GF was estimated using the ΔΔCt method (Livak and Schmittgen, 2001), where the accumulation of RNA2 and RNA3 are calculated relative to the accumulation of RNA1. Primer amplification efficiencies were shown to be optimal and equal in separate RT-qPCR runs.

Because qPCR is considered our golden standard, we performed an additional control experiment to validate the qPCR assay. We spiked 1:100 diluted cDNA of a mock inoculated *Nicotiana tabacum* plant with known ratios of amplicons of each CMV segment. The CMV amplicons used for spiking were purified from a PCR reaction using the GeneJET PCR purification kit (Thermo Scientific) following the manufacturers instructions. The sample used was a *N. tabacum* plant inoculated with CMV isolate I17F in a separate experiment. Four different ratios of RNA1:RNA2:RNA3 were tested: 1:1:1, 1:10:10, 10:1:10 and 10:10:1. Here, a ratio of 1 and 10 correspond to 10^5^ and 10^6^ copies per PCR well, respectively. Thermocycling was conducted as described above. Estimates of segment frequencies were made using the ΔΔCt method (Livak and Schmittgen, 2001) where the fold-change of high frequency segments was determined relative to that of low frequency segments to show the known segment ratios could be reconstituted from the qPCR Ct values.

### 2.5 Digital PCR

The QIAcuity dPCR (Qiagen) with 96-well nanoplate generating 8500 partitions per well was used for dPCR GF quantification. We designed an EvaGreen-based assay (Qiagen) using the same primer pairs as in the qPCR. Each cDNA sample was diluted 1000-fold and assayed in duplo for RNA1, RNA2 and RNA3 each. Using 1000-fold dilution ensured we were in the dynamic range of the dPCR for the high titer samples. Cycling consisted of initial denaturation at 95 °C for 2 min, followed by forty cycles of 95 °C for 15 s 60 °C for 15 s, and 72 °C for 15 s, and a final step of 40 °C for 5 min. The GF was estimated using the following calculation within the QIAcuity analysis software:

$$\lambda =\;\frac{-ln\;\left(\frac{Pv\;-\;Pp}{Pv}\right)}{V}$$

Where *V* is partition volume in µl, Pv is the numer of valid partitions, Pp is the number of positive partitions, and *λ* is the copies of target per µl.

### 2.6 Illumina RNA sequencing

1000 ng of total RNA was sequenced using Illumina NovaSeq (150 bp paired ends). Library prep was done using the Illumina TruSeq stranded mRNA kit at Macrogen Europe BV (Amsterdam, the Netherlands). During the library prep, no enrichment of poly-A tailed RNA or depletion of ribosomal RNA was performed to ensure the library was constructed from the same material as the Nanopore library. Six Gigabases of data were generated for each sample. GFs were estimated based on coverage after mapping the reads onto the CMV subgroup I (strain Fny) reference genome. Strain I17F used in our experiment has over 99% nucleotide similarity to strain Fny.

### 2.7 Nanopore direct RNA sequencing

Direct RNA Nanopore sequencing chemistry requires the presence of a poly-A tail to ligate the sequencing adapter. Some plant viruses, including CMV, do not have poly-A tails. Therefore, a poly-A tailing step was necessary prior to sequencing. For each RNA sample, approximately 1000 ng of RNA was poly-A tailed. Briefly: to a thin walled PCR tube, 15 µl RNA (final concentration 50 ng/µl), 2 µl 10X *E. coli* Poly(A) Polymerase Reaction Buffer, 2 µl ATP (10 mM, final concentration 1 mM) and 1 µl (5 units) *E. coli* poly(A) polymerase (NEB) were added, to a total of 20 µl. The reaction was incubated for 30 m at 37 °C and was stopped by AMPure RNA bead cleanup.

For sample cleanup prior to library preparation, 70 ul AMPure RNA beads were added to 20 ul polyA RNA in a lo-bind eppendorf tube. This sample was agitated on the hula mixer for 5 m at room temperature to allow the RNA to bind to the beads. The beads were then pelleted on a magnetic rack. The supernatant was pipetted off, and while keeping the tube on the magnet, freshly prepared 70% EtOH was added to the tube without disturbing the pellet. The eppendorf was rotated 180° to allow the pellet to migrate to the other side of the tube. This step was repeated, and the EtOH was pipetted off. The beads were then resuspended in 20 ul RNAse free water for 5 min at room temperature and subsequently pelleted again on the magnet. The eluate containing the RNA was transferred into a new, lo-bind eppendorf tube. Concentration and purity were measured on the Nanodrop. When the concentration was sufficient (> 5 ng/ul), we proceeded immediately with RNA library preparation (ONT kit SQK-RNA002) following the manufacturer's instructions. Multiple samples (up to three) were run per MinION flow cell successively with a wash step in between samples (using ONT kit EXP-WSH004) to prevent cross contamination between libraries. Ideally, we would have used barcodes to multiplex several samples on a single flow cell, but this currently is not possible for RNA sequencing experiments with ONT. For further downstream bioinformatic analysis, only Nanopore reads with a mean Phred score >7 were selected.

### 2.8 Bioinformatic analyses of the HTS data

For the HTS data generated by either Illumina or Nanopore sequencing, GFs were estimated based on coverage after mapping the reads onto the CMV subgroup I (strain Fny) reference genome (accessions NC_002034.1, NC_002035.1 and NC_001440.1). Bioinformatics analyses for the HTS data were performed in CLC Genomics Workbench v22 (Qiagen). Reads were trimmed and mapped to the CMV-Fny reference genome using default settings. All parameters used can be found in the supplementary materials (Table S3). Read coverage along each genome segment was exported in TSV format and imported into R for further analysis. For Illumina, coverage across the genome is uneven due to amplification bias in the library preparation (Fig. S2). To account for this, we use the geometric mean coverage as the measure of relative segment accumulation. The geometric mean is less sensitive to outliers than the arithmetic mean. For Nanopore, there is no amplification step in the sequencing protocol. However, there is a strong 3′ sequencing bias (Wongsurawat et al., 2019) since sequencing adapters for Nanopore dRNA sequencing attach to the poly-A tail located at the 3′ end of the RNA and reads are only generated from this end. Therefore, any RNA degradation or fragmentation will result in a drop in coverage towards the 5′ end. If we were to use the average coverage as a GF estimate, longer genome segments (RNA1) would be underestimated whereas shorter genome segments (RNA3) would be overestimated. Therefore, we used the maximum coverage detected as our measure of segment accumulation (Fig. S1).

### 2.9 Comparison of genome formulas across methods and samples

The GF is determined either relative to the least abundant segment or as relative accumulation (Sicard et al., 2013) (i.e. $f_{i\;}=1$, where *f* is the relative frequency of the *i^th^* segment). We use the relative accumulation for analyses to ensure that observed differences between the accumulation of multiple segments are not influenced by differences in the segment used for normalization.

### 2.10 Variation in genome formula estimates between methods: Δ*M*

The Δ*GF* is a metric for comparing virus accumulation taking into account all segments of a multipartite virus (Sicard et al., 2013). The Δ*GF* takes the absolute distance between relative frequencies and sums this across segments. Here we introduce theΔ*M*: a metric which adapts the Δ*GF* to perform a pairwise comparison of genome formulas between methods. We take the Δ*M* to be the sum of the absolute difference between *f_i_* for two methods (e.g., *m1* or *m2*) for each biological replicate (*j*), divided by 2:

$\Delta M_{j}\;=\;\sum_{i=1}^{3}\left|\frac{f_{i,j,m1}\;-\;f_{i.j,m2}\;}{2}\right|\;$.

The Δ*M* metric allows us to directly compare GF estimates between two methods, by comparing the relative accumulation within biological replicates across methods. Thus, the only variation when comparing Δ*M* within a replicate should stem from the method itself and includes differences that can be attributed to both systematic bias and random variation.

### 2.11 Models of genome formula variation: ΔGF

As there may be substantial noise in the GF estimates, it is valuable if we can determine where this noise originates from. We propose that the observed variation can be random, host derived, method derived, or by the interaction between the host and the method. To that end, we designed four models describing the variation in the GF.

Under the most simple assumption, there is one mean GF which is not significantly affected by the host species or the GF estimation method. This model is our null model. Alternatively, there can be an effect of the host species on the GF estimate. In this model, referred to as ‘H’, there is a host species specific mean GF (three means). Another possibility is that there is an effect of method on the GF estimate due to a systematic bias in quantification. In model ‘M’, there is a method-specific mean GF (four means). Lastly, the interaction between host species and the quantification method may affect the GF estimate. In model ‘HM’ there is a host and method specific mean GF (12 means). To compare these models, we calculated the Δ*GF* relative to the mean GF value for the ith segment ($\underline{\;f_{i}}$) per biological replicate (*j*) of all samples (null), the mean GF per host species (H), the mean GF per method (M), or the mean GF per host species per method (HM):

$\Delta GF_{j}\;=\;\sum_{i=1}^{3}\left|\frac{f_{i,\;j}\;-\underline{\;f_{i}}\;}{2}\right|\;$.

To consider how well supported the different models are, we determined the residual sum of squares (RSS) for each fitted model, and then calculated the negative log likelihood (NLL) as $NLL=\frac{n}{2}ln\left(\frac{RSS}{n}\right)$, where *n* is the number of observations (Johnson and Omland, 2004). This approximation allowed us to determine the Akaike information criterion (AIC) (Akaike, 1974) for each model and perform model selection.

## 3 Results

We measured the genome formula (GF) (i.e. relative accumulation of genome segments) for CMV using four different methods and three different hosts, with the aim of determining how congruent the GF estimates are across methods and providing recommendations of which method is applicable to a given biological question or system.

### 3.1 Genome formulas are similar across hosts and methods

**Fig. 1:**
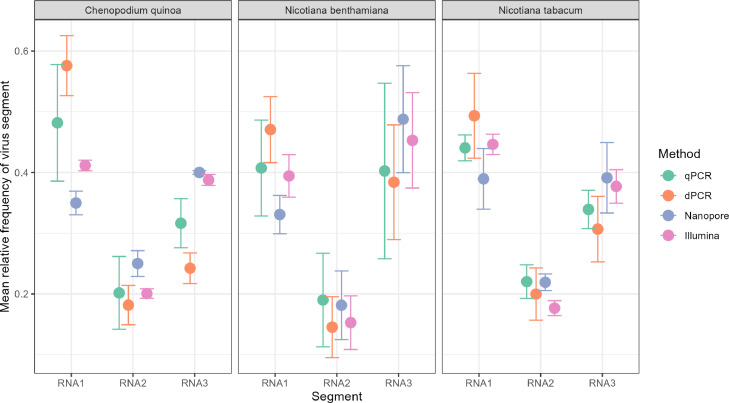
GF estimates across the different methods. The y-axis shows relative accumulation of segment RNA1, RNA2 or RNA3. Methods are indicated by color. Each point shows the mean relative segment frequency for four biological replicates. Error bars represent standard deviation (N=4).

The overall mean GF of CMV across hosts and methods is approximately 0.43 : 0.19 : 0.37 (RNA1 : RNA2 : RNA3). Generally, the accumulation of RNA2 is lowest, RNA3 is intermediate and RNA1 is at the highest frequency. Interestingly, the PCR-based methods tend to estimate higher frequency of RNA1, whereas the sequencing-based methods tend to estimate higher frequencies of RNA3 (Fig. 1). Unlike prior work in faba bean necrotic stunt virus (FBNSV) (Sicard et al., 2013) and alfalfa mosaic virus (AMV) (Wu et al., 2017), a host-specific GF between three tested plant species is not observed (Table 2). A complete overview of genome formula estimates across hosts and methods can be found in Table S1 and Fig. S3.

**Table 2:** Overview of model fit. Negative log likelihood (NLL) and Akaike information critereon (AIC) are shown. Akaike weights indicate the relative likelihood of each model compared to the other models in this set.

Model	Factors included	Number of parameters in model (K)	Mean dGF (± CI)	NLL	AIC	Akaike weights
null	–	2	0.0826 ± 0.0155	−111.5	−219.0	0.0231
H	host species	6	0.0788 ± 0.0126	−115.7	−219.4	0.0288
M	method	8	0.0626 ± 0.0146	−121.2	−226.4	0.9456
HM	host species and method	24	0.0538 ± 0.0107	−131.2	−214.5	0.0025

We consider qPCR the ‘golden standard’ method for GF estimation, as this method is most widely used. Therefore, additional qPCR validation was performed using four samples spiked with known ratios of each RNA, varying 10-fold in accumulation. Segment frequencies could be retrieved reliably based on the known input quantities, as shown in Fig. S4. When we performed linear regression of the known and measured relative frequencies, this resulted in model parameters (slope = 1.031 ± 0.079, intercept = 0.010 ± 0.030) very close to the expected values for a perfect correlation (slope = 1, intercept = 0) and a high coefficient of determination (*r*^2^ = 0.944), confirming the validity of these measurements.

### 3.2 Correlation between relative accumulation estimates

**Fig. 2:**
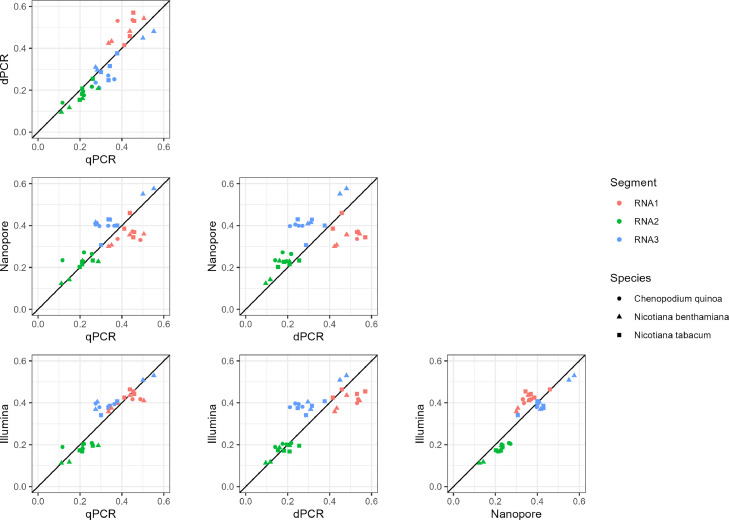
Congruence between relative accumulation estimates for each method. Colors indicate the RNA segment and shapes represent the host species. In each plot, relative frequencies are plotted against each other for different methods. The 1:1 line is shown diagonally in each plot.

If the GF estimates resulting from each method are highly congruent, a strong correlation is expected between the relative accumulation measured between the methods. We use pairwise correlations to plot relative accumulation per segment for each method to determine patterns in the GF (Fig. 2). Nanopore sequencing underestimates RNA1 accumulation, and overestimates RNA3 accumulation compared to all other methods. The opposite is seen in the dPCR, where RNA1 accumulation is systematically overestimated in comparison to other methods (Fig. 2).

**Fig. 3:**
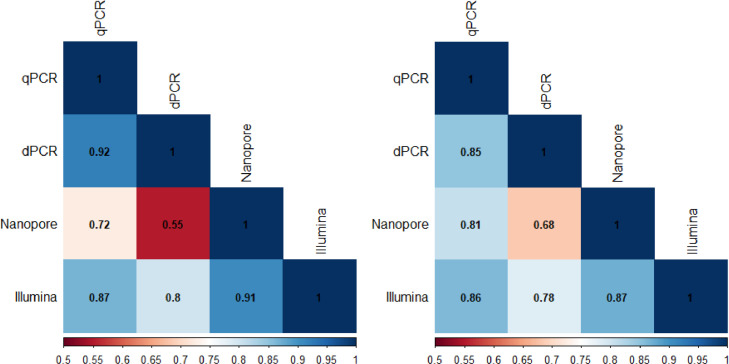
Correlation plots for coefficient of correlation (r) (left) and *scaled*Δ*M* (right). To ensure r and Δ*M* values are comparable, the Δ*M* was rescaled to range from 0 to 1, where 1 is a perfect match between methods. The scaling was done as follows: scaledΔM=1−2·ΔM.

To compare GF estimates across methods, we used both Pearson's coefficient of correlation (r) and an adaptation of the ΔGF (Sicard et al., 2013): the ΔM (Fig. 3). The coefficient of correlation quantifies the correlation in relative accumulation of segments between methods in a pairwise manner, and ranges from zero (no congruence) to one (completely congruent). The ΔGF metric takes into consideration that we are not merely interested in the accumulation of each segment, but also in the ratio between the segments. The ΔGF reduces the GF, a ratio between *n* segments, to a single number. Here, we have adapted the ΔGF metric (Sicard et al., 2013) to perform a pairwise comparison between methods for each biological replicate: the ΔM. Together, the r and ΔM values give an indication of how congruent methods are for assessing the GF of a multipartite virus. Both the r and the ΔM show similar trends (Fig. 3). Notably, Nanopore has the lowest similarity to other methods and is especially different from the dPCR (r = 0.55, ΔM=0.68) (Fig. 3). Both PCR-based methods (dPCR and qPCR) are congruent with each other (r and ΔM>0.85), as are the sequencing-based methods (Nanopore and Illumina, r and ΔM>0.87). Due to observations not being independent, p-values are not reported here.

### 3.3 Models of genome formula variability

Variability in the GF estimates can result from various factors. Since there is substantial noise in our GF estimates, we devised four models in which we attribute the variation in GF estimates to different possible factors: host (H), method (M), both host and method (HM), or neither (null). Across these models, we calculate a ΔGF relative to the mean(s) of each factor in the model. The mean ΔGF, in this context, is indicative of model fit. A higher mean ΔGF for a model indicates that there is high GF variability compared to the mean. A lower mean ΔGF indicates that there is lower variability of the GF around the mean for a model, indicating a better model fit.

Our results show the effect of method on the GF is strong, with the lowest AIC out of all the models and an Akaike weight of >0.9 (Table 2). Results therefore indicate that variability in GF estimates between methods is substantial. By contrast, the effect of host on the GF is negligible with an AIC and Akaike weight close to the null model. Model HM which incorporates both host and method is overparameterized, with a low NLL but a high AIC. Note that the absolute value of the dGF residual sum of squares is low, and therefore both NLL and AIC values are negative.

## 4 Discussion

We performed a systematic comparison of established and novel methods for quantifying the genome formula (GF) of a multipartite plant virus in three different hosts. Overall, all methods reported similar genome formulae and the genome formula was similar across hosts. The qPCR method correlates strongly with the dPCR and correlates reasonably with quantification using Illumina short-read sequencing. Correlation between Nanopore long read sequencing-based quantification and the PCR-based methods was low, though correlation between both sequencing-based methods (Nanopore and Illumina) was high. We observed some systematic, method-specific biases in GF quantification. Notably, dPCR systematically overestimates RNA1 accumulation, whereas Nanopore systematically underestimates RNA1 and overestimates RNA3 accumulation. A possible explanation for the overestimation of RNA3 observed for Nanopore may be caused by the presence of subgenomic RNA. The 3′ ends of subgenomic RNAs from positive-strand viruses are identical to those of genomic RNA, but the 5′ ends have been deleted to place the 5′ end of the RNA close to the start codon of the ORF, allowing for translation of ORFs downstream of another ORF encoded by the same genomic segment (Miller and Koev, 2000). CMV has a subgenomic RNA4 (sgRNA4) of 1031 bp long which encodes the coat protein. Prior work using Northern blot hybridization and RT-qPCR has shown RNA3 and sgRNA4 accumulate to an approximately equal number of copies (Feng et al., 2006). Because the nucleic acid sequence of sgRNA4 is identical to that of genomic RNA3, sequencing cannot distinguish between the two. For the PCR-based methods, primers were designed outside of the subgenomic region. However, for the Nanopore estimate we take the maximum coverage which lies towards the 3′ end of the genome segment that also comprises the sgRNA4. Nanopore dRNA sequencing is known to have a strong coverage bias towards the 3′ end (Wongsurawat et al., 2019). Using this method, it was not possible to differentiate sgRNA from gRNA *in silico*, as there is no clear drop in sequencing coverage at the 5′ end of sgRNA4 when reads are aligned to RNA3. This may be overcome by sequencing cDNA generated by random primers as described by (Liefting et al., 2021). It should be noted that CMV subgenomic RNA4A (sgRNA4A) may also be present, though we expect little effect of this segment on the GF estimate due to its low accumulation compared to RNA2 (Palukaitis and García-Arenal, 2019).

GFs in virus species belonging to the *Bromoviridae* family*,* such as CMV and AMV, appear to show smaller host-dependent differences than for FBNSV, a member of the *Nanoviridae* (Sicard et al., 2013; Wu et al., 2017). When GF differences are larger, as is the case for FBNSV, we expect the choice of method to have a smaller effect on the GF estimate than the host. Therefore, we expect comparing GFs obtained using different methods may be feasible for some multipartite viruses, if the magnitude of the difference in GF is large. Method specific GF differences have been found for FBNSV when comparing qPCR and HTS analysis (consisting of rolling circle amplification (RCA) followed by Illumina sequencing). The differences between HTS and qPCR were small, but significant and stemmed from variation introduced during the RCA step and the Nextera library prep (Gallet et al., 2017). Newer Illumina kits -such as the Illumina DNA prep- may not have this problem.

The GF is one of the recently hypothesised benefits of the multipartite genome organization of viruses, and as such has been the subject of investigation across systems ranging from experimental to ecological. The preferred method for GF quantification depends on the research question and experimental context.

For example, when exploring GF variation experimentally using a model system, establishing and validating a PCR-based quantification assay is a worthwhile investment. Examples of such experiments are testing for the effect of biotic (such as the soil biome, presence of other pathogens) and abiotic (such as soil nutrients, drought, light and temperature) effects on the GF. Whilst a well-designed qPCR is an effective tool for GF quantification, testing of the amplification efficiencies and dynamic range is essential and must not be overlooked. For a tripartite ssRNA virus such as CMV, we estimate that the cost of RT-qPCR per sample is currently around €13. However, this cost rises with the number of segments this can increase to over €20 per sample for an octapartite virus such as FBNSV. dPCR has the advantage over qPCR of smaller error estimates, wider dynamic range at low template concentrations and absolute quantification of viral titer. For detecting subtle changes in the GF, dPCR may therefore be preferable over qPCR. Additionally, dPCR allows for multiplexing of up to four or five targets in a single well. For multipartite viruses with between 2 and 5 genome segments, this means all segments could be quantified in a single well, reducing costs and increasing throughput. The costs for dPCR was €27 per sample in this study -due to our use of technical replicates- but can be reduced to <€5 per sample if multiplex probes are used.

Conversely, ecological studies may not benefit from a PCR-based quantification approach. Natural habitats are teeming with undiscovered multipartite viruses and virus isolates, whose genomes will be divergent from known isolates. For a set of diverse, natural virus isolates, the development of a quantitative or digital PCR assay is laborious due to the need to first sequence the genomes and subsequently design and validate primers for each viral genome segment. In those systems, the GF may be inferred directly from metagenomics HTS sequencing data. Currently, there is little known about the ecological features associated with multipartite viruses, and to our knowledge no direct estimates of GFs measured in field systems exist. An exploration of GF variability using HTS data would thus have caveats in terms of accuracy and comparability to existing GF studies which are mostly PCR-based, but could overall contribute immensely to the gap in knowledge about GF variation in natural systems. Table 3 summarises the pros and cons of using each of the four tested methods for GF quantification.

**Table 3:** Pros and cons of the use of four different methods for GF estimation.

Method	Pros	Cons
RT-qPCR	•Relatively affordable •Readily available protocols and tools Scalable for small project size	•Requires sequence knowledge of virus target •Amplification efficiencies need to be equal for some analysis methods •Relative quantification •Lower dynamic range •Large confidence interval when the virus titer is low
RT-dPCR	•Absolute quantification •Possibilities for multiplexing •Quantification is possible at low virus titer	•Requires sequence knowledge of virus target •Expensive equipment investment •Consumables costs higher than conventional qPCR •Chance of oversaturation at high virus titer, one may need to run a qPCR first, or have multiple runs, to make sure you are in the right window for dilutions. This will cost more time and drive up the cost.
Illumina RNA sequencing	•Untargeted (i.e. does not depend on a reference genome, as the viral genome can be assembled from the data)	•Uneven amplification along genome Relatively expensive
Nanopore dRNA sequencing	•Untargeted (i.e. does not depend on a reference genome, as the viral genome can be assembled from the data)	•dRNA sequencing has a strong 3′ prime coverage bias Laborious Relatively expensive

Finally, our results suggest that there are different biases associated with the methods tested. The similarity between methods based on the same approach (i.e., PCR-based vs. HTS-based methods) suggests that some of these biases may be systematic and driven by the approach. Ideally, GFs calculated by different methods should therefore not be compared without validation, and in general such comparisons should be approached cautiously.

## Data availability

Sequencing data is available at NCBI SRA BioProject PRJNA927308. qPCR and dPCR data are available *via* Dryad: https://doi.org/10.5061/dryad.b8gtht7gc. Data analysis scripts and modeling code have been made available *via* Zenodo: https://doi.org/10.5281/zenodo.7568957.

## Funding

MJ, AG, and MZ were supported by a VIDI grant from The Netherlands organization for Scientific Research (NWO 016.Vidi.171.061) to MZ.

## CRediT authorship contribution statement

**Dieke Boezen:** Conceptualization, Methodology, Investigation, Formal analysis, Visualization, Writing – original draft. **Marcelle L Johnson:** Conceptualization, Methodology, Writing – review & editing. **Alexey A Grum-Grzhimaylo:** Methodology, Formal analysis, Writing – review & editing. **René AA van der Vlugt:** Resources, Writing – review & editing. **Mark P Zwart:** Supervision, Formal analysis, Funding acquisition, Writing – review & editing.

## Data Availability

Data analysis scripts and modeling code have been made available via Zenodo: https://doi.org/10.5281/zenodo.7568957.

## Appendix Supplementary materials

Image, application 1
