# Supplementary material for: Evaluation of sequencing and PCR-based methods for the quantification of the viral genome formula
Source: Virus Res. 2023 Feb 7;326:199064. doi: 10.1016/j.virusres.2023.199064 (PMC10194290; doi:10.1016/j.virusres.2023.199064)
Supplement: Supplementary file 1 [file mmc1.docx]

### Supplementary tables

**Table S1:** Genome formula estimates, summarized across species and methods. Means and standard deviations are shown for the relative accumulation of each segment. The mean estimate is based on four biological replicates per host per method (N=4). The mean GF for ‘all species’ is based on twelve replicates (four per host, N=12). The mean across methods for all species is based on all data (N=48).

|  |  | **GF Mean** | | |  | **GF SdDev** | | |
| --- | --- | --- | --- | --- | --- | --- | --- | --- |
| **Method** | **Host species** | **RNA1** | **RNA2** | **RNA3** |  | **RNA1** | **RNA2** | **RNA3** |
| qPCR | *Chenopodium quinoa* | 0.48 | 0.20 | 0.32 |  | 0.10 | 0.06 | 0.04 |
|  | *Nicotiana tabacum* | 0.44 | 0.22 | 0.34 |  | 0.02 | 0.03 | 0.03 |
|  | *Nicotiana benthamiana* | 0.41 | 0.19 | 0.40 |  | 0.08 | 0.08 | 0.14 |
|  | All species | 0.44 | 0.20 | 0.35 |  | 0.07 | 0.05 | 0.09 |
| dPCR | *Chenopodium quinoa* | 0.58 | 0.18 | 0.24 |  | 0.05 | 0.03 | 0.03 |
|  | *Nicotiana tabacum* | 0.49 | 0.20 | 0.31 |  | 0.07 | 0.04 | 0.05 |
|  | *Nicotiana benthamiana* | 0.47 | 0.15 | 0.38 |  | 0.05 | 0.05 | 0.09 |
|  | All species | 0.51 | 0.18 | 0.31 |  | 0.07 | 0.05 | 0.08 |
| Illumina | *Chenopodium quinoa* | 0.41 | 0.20 | 0.39 |  | 0.01 | 0.01 | 0.01 |
|  | *Nicotiana tabacum* | 0.45 | 0.18 | 0.38 |  | 0.02 | 0.01 | 0.03 |
|  | *Nicotiana benthamiana* | 0.39 | 0.15 | 0.45 |  | 0.03 | 0.04 | 0.08 |
|  | All species | 0.42 | 0.18 | 0.41 |  | 0.03 | 0.03 | 0.06 |
| Nanopore | *Chenopodium quinoa* | 0.35 | 0.25 | 0.40 |  | 0.02 | 0.02 | 0.00 |
|  | *Nicotiana tabacum* | 0.39 | 0.22 | 0.39 |  | 0.05 | 0.01 | 0.06 |
|  | *Nicotiana benthamiana* | 0.33 | 0.18 | 0.49 |  | 0.03 | 0.06 | 0.09 |
|  | All species | 0.36 | 0.22 | 0.43 |  | 0.04 | 0.04 | 0.07 |
| All methods | All species | 0.43 | 0.19 | 0.37 |  | 0.08 | 0.05 | 0.09 |

**Table S2**: Overview of primers used for quantification of CMV in qPCR and dPCR. Positions relative to CMV Fny refers to accessions NC_002034.1, NC_002035.1 and NC_001440.1 specifically.

| **Target** | **Primer name** | **Primer sequence** | **Amplicon length** | **Annealing temperature** | **Position relative to CMV Fny** |
| --- | --- | --- | --- | --- | --- |
| CMV RNA1 | cmvrna1a_1f | GCACAACCCGTGAGTGAGG | 83 | 60 °C | 1706-1724 |
| CMV RNA1 | cmvrna1a_1r | TCCCTTCCACAAACATCAGCAG |  | 60 °C | 1767-1788 |
| CMV RNA2 | cmvrna2a_1f | GGTGTTGTTGATAATGCGACTCTG | 94 | 60 °C | 789-812 |
| CMV RNA2 | cmvrna2a_1r | CGATGGTTGGCGTTGGACAT |  | 60 °C | 863-882 |
| CMV RNA3 | cmvrna3a_1f | ACCATGATCTTCCCGCTTTGG | 91 | 60 °C | 511-531 |
| CMV RNA3 | cmvrna3a_1r | ACGACAGCAAAACACCGCTT |  | 60 °C | 582-601 |

**Table S3:** Overview of parameter settings used for mapping of Illumina reads to the CMV Fny reference genome.


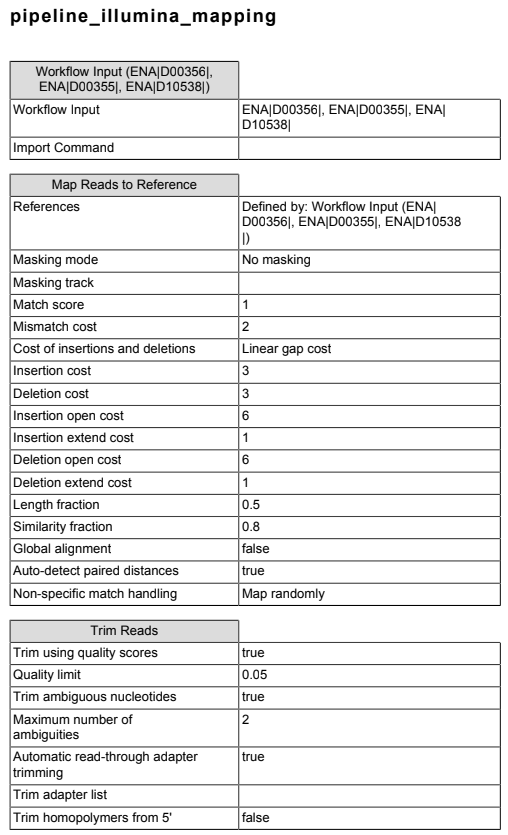


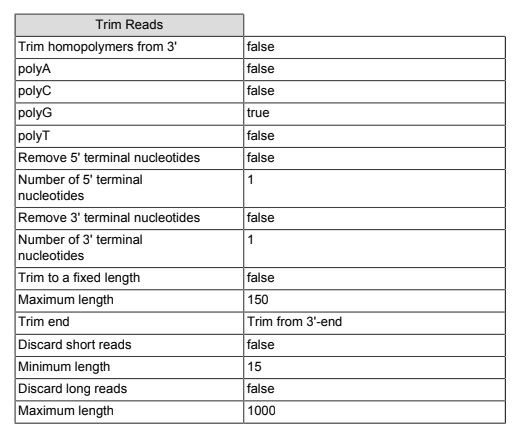


**Table S4:** Overview of parameter settings used for mapping of Nanopore reads to the CMV Fny reference genome.


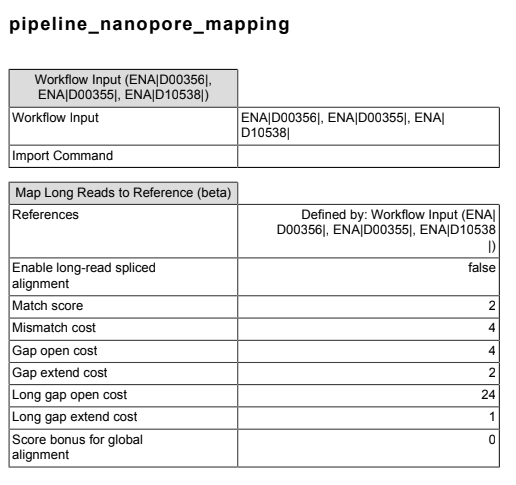


### Supplementary figures

**Figure S1:** Nanopore coverage across the genome for each segment. The x-axis shows the position along the genome (bp). The y-axis shows the coverage at that position according to the reference-based mapping. Note the 3’-end coverage bias.
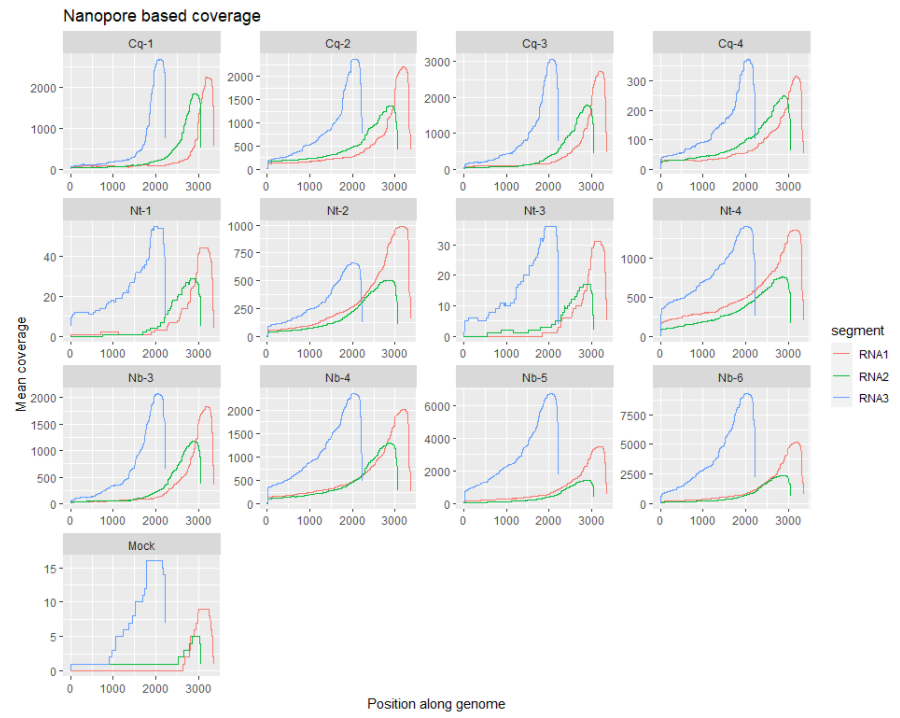


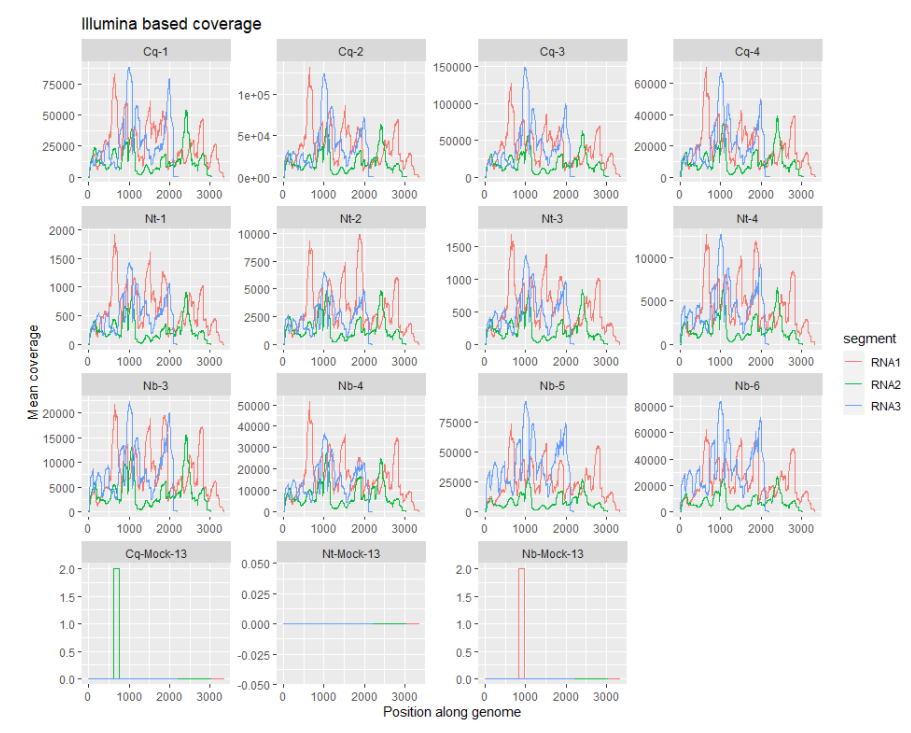
**Figure S2:** Illumina coverage across the genome for each segment. The x-axis shows the position along the genome (bp). The y-axis shows the coverage at that position according to the reference-based mapping.


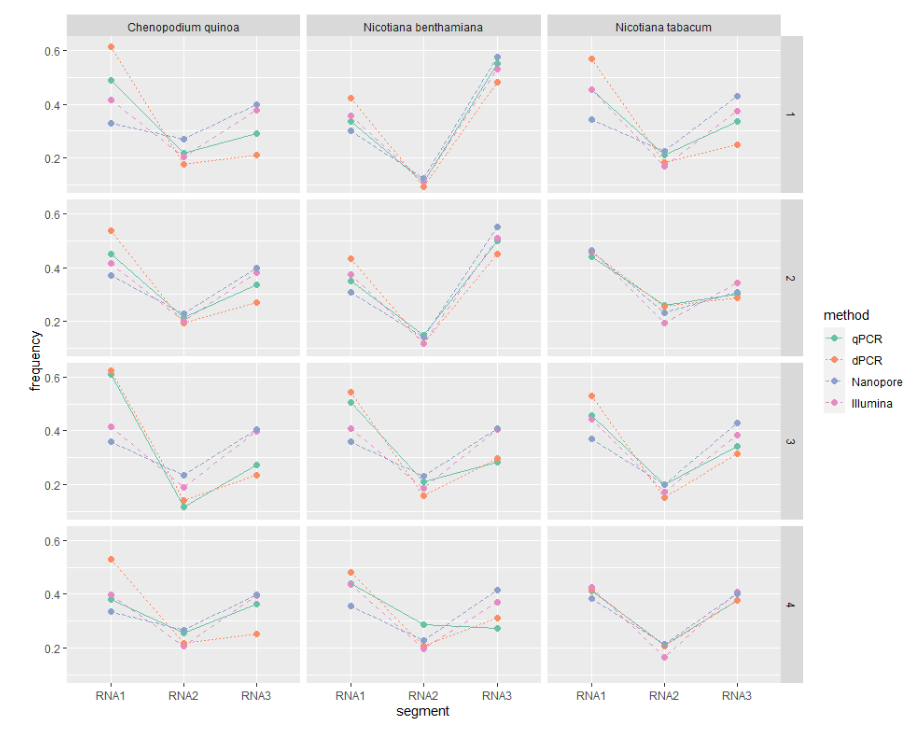


**Figure S3:** GF estimates across each sample for the different methods. The y-axis shows relative accumulation of segment RNA1, RNA2 or RNA3. Methods are indicated by line type and color. Each box indicates a different biological replicate, and for RT-qPCR the mean value of the three technical replicates is shown.


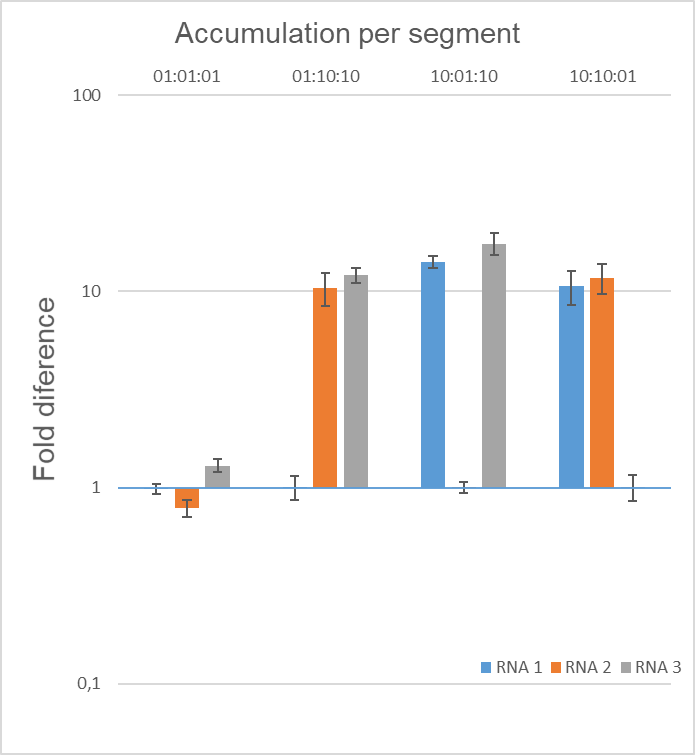


**Figure S4:** Validation of qPCR using spiking. Amplicons of RNA1, RNA2 and RNA3 were spiked in four known ratios: 1:1:1 (equimolar), 1:10:10, 10:1:10 and 10:10:1 (where one segment is at low frequency, two at high frequency). The ratios used for spiking are shown at the top of the graph. The fold difference relative to the mean of the low frequency segment(s) is shown. Error bars indicate the 95% confidence intervals calculated from three technical replicates. RNA1 is shown in blue, RNA2 in orange, and RNA3 in grey. The input ratios are reproduced reliably, with estimates close to the expected quantities.
